# Three-Dimensional Reconstruction with a Laser Line Based on Image In-Painting and Multi-Spectral Photometric Stereo †

**DOI:** 10.3390/s21062131

**Published:** 2021-03-18

**Authors:** Liang Lu, Hongbao Zhu, Junyu Dong, Yakun Ju, Huiyu Zhou

**Affiliations:** 1 College of Information Science and Engineering, Ocean University of China, Qingdao 266100, China; luliang@stu.ouc.edu.cn (L.L.); zhuhongbao@stu.ouc.edu.cn (H.Z.); juyakun@stu.ouc.edu.cn (Y.J.); 2 School of Informatics, University of Leicester, Leicester LE1 7RH, UK; hz143@leicester.ac.uk

**Keywords:** image in-painting, generative adversarial network, multi-spectral photometric stereo, and laser extraction

## Abstract

This paper presents a multi-spectral photometric stereo (MPS) method based on image in-painting, which can reconstruct the shape using a multi-spectral image with a laser line. One of the difficulties in multi-spectral photometric stereo is to extract the laser line because the required illumination for MPS, e.g., red, green, and blue light, may pollute the laser color. Unlike previous methods, through the improvement of the network proposed by Isola, a Generative Adversarial Network based on image in-painting was proposed, to separate a multi-spectral image with a laser line into a clean laser image and an uncorrupted multi-spectral image without the laser line. Then these results were substituted into the method proposed by Fan to obtain high-precision 3D reconstruction results. To make the proposed method applicable to real-world objects, a rendered image dataset obtained using the rendering models in ShapeNet has been used for training the network. Evaluation using the rendered images and real-world images shows the superiority of the proposed approach over several previous methods.

## 1. Introduction

Three-dimensional reconstruction has been one of the active research areas in computer vision for several decades. There have been many algorithms that can perform high-precision 3D reconstruction of the target, including laser scanning technology [1,2,3], photometric stereo [4], structure from motion [5], multi-view stereo [6], etc. There is a recent trend that attempts to combine deep learning with geometry-based 3D reconstruction methods e.g., photometric stereo. However, many issues exist when such 3D reconstruction methods are used in an uncontrolled environment, e.g., underwater 3D imaging: (1) Image acquisition is difficult, while deep learning usually requires a large amount of data for training; (2) The images required by photometric stereo algorithms should be obtained when the camera and the target are relatively still, while it is difficult to achieve in an underwater environment; (3) The result obtained by the photometric stereo algorithm does not have scale accuracy, that is, only the shape is relatively accurate, and the height information is not accurate.

In order to solve the first issue, we can use the rendered images as the training set [7]. For the second issue, we could use a multi-spectral photometric stereo algorithm [8,9,10], which only needs a single-color image [11,12,13,14]. Finally, for the third issue, we could add a laser line on the RGB image, and correct the result of the multi-spectral photometric stereo algorithm through the height information of the pixels on the laser line [15,16].

However, in the process of 3D reconstruction using multi-spectral images with laser lines, there are the following problems: (1) The laser line will pollute the imaging result of the color light source, that is, the area covered by the laser light source will lose pixel information; (2) The color light source will influence the result of laser line extraction.

Inspired by the strong ability of Generation Adversarial Networks (GAN) to model the data distribution, in this study, we use GAN based on image in-painting to solve the above issues. Through the improvement of the network proposed by Isola [17], we proposed a network which can separate the multi-spectral image with a laser line into a clean laser image and an uncorrupted multi-spectral image without the laser line. And based on the proposed network and Fan’s [16] algorithm, we achieved accurate 3D reconstruction using a multispectral image with a laser line.

**Figure 1:**
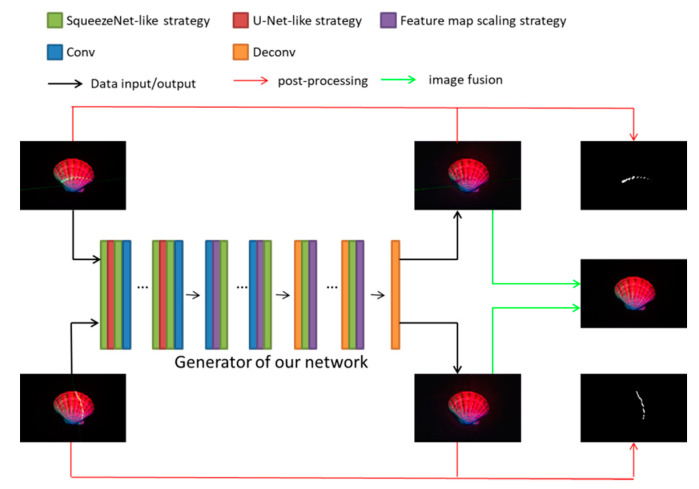
The overview of the proposed method. Input images are illuminated with red, green and blue lights simultaneously together with a projected laser line. We propose a network based on Generation Adversarial Networks (GAN) and image in-painting to separate the multi-spectral image with a laser line into a clean laser image and an uncorrupted multi-spectral image without the laser line. The details of the three strategies represented by green, red and purple will be introduced in Section 3.1, and the post-processing represented by the red and green arrows will be introduced in Section 4.2.

The overview of the proposed method is shown in Figure 1.

The main contributions of this article are summarized as follows:

(1) Through the improvement of the network proposed by Isola [17], we propose a Generative Adversarial Network based on image in-painting to realize the effective estimation of the pixel values at the locations covered by the laser line in the multi-spectral image;
(2) The proposed network can effectively extract the laser line in the multi-spectral image;
(3) Through adding certain U-Net-like structures to the generator of GAN, the proposed network can produce stable results;
(4) Based on the proposed network and Fan’s [16] algorithm, we achieve accurate 3D reconstruction using a multispectral image with a laser line.

The remaining of this article is organized as follows: Section 2 introduces the related work; Section 3 introduces the network structure, related parameters and training process of the Generative Adversarial Network proposed in this article; Section 4 introduces the rendered image dataset used in this article and the results of the rendered images and the real images; Section 5 concludes this paper.

## 2. Related Work

In this section, we firstly make an introduce on the traditional multi-spectral photometric stereo in Section 2.1, and then make an introduce on photometric stereo algorithm based on laser line correction in Section 2.2, finally, we briefly review some traditional methods on laser line extraction algorithms in Section 2.3, and some image in-painting algorithms based on deep learning in Section 2.4.

### 2.1. Introduction of Multi-Spectral Photometric Stereo

The traditional multi-spectral photometric stereo technique can reconstruct the 3D geometry with a color image. The image should be obtained under the trichromatic light source with known angles.

The principle of multi-spectral photometric stereo is shown in Equation (1).

(1) $$c_{i}\left(x,y\right)=\underset{i}{\sum }l_{j}^{T}n\left(x,y\right)\int E_{j}\left(\lambda \right)R\left(x,y,\lambda \right)S_{i}\left(\lambda \right)d\lambda$$

where, *l_j_* is the *j*-th illumination direction vector, *n*(*x*, *y*) is the normal vector of a certain point of the target, *E_j_*(λ) is the illumination intensity, *R*(*x*, *y*, λ) is a parameter related with the albedo and chromaticity of a certain point of the target, and *S_i_*(λ) is the color response of the camera photosensitive element.

Assume *R*(*x*, *y*, λ) as the product of *ρ*(*x*, *y*) and *α*(λ), which represent the albedo and the chromaticity respectively, then combining all the terms which are related with λ as a whole, and we can get a parameter matrix *V*, as shown in Equation (2).

(2) $$V_{ij}=\int E_{j}\left(\lambda \right)\alpha \left(\lambda \right)S_{i}\left(\lambda \right)d\lambda$$

So we can rewrite Equation (1) as Equation (3), and obtain Equation (4).

(3) C=VLρn

(4) $$n=\frac{V^{-1}L^{-1}c}{∥V^{-1}L^{-1}c∥}$$

That is, the exact solution of the normal vector of the target surface can be obtained on the premise that the target’s chromaticity and the illumination direction are known.

However, these formulas are based on images acquiring in an ideal state. Since the chromaticity of each point on the surface of the target are unpredictable, and meanwhile, the near-field light source, instead of the parallel light source, is used in practical applications, which is assumed in the traditional multi-spectral photometric stereo algorithm, the direction and the intensity of the light sources are also different for each point on the surface of the target. That is, in formula (2), the parameter matrix $V_{ij}$ of each piont are different cause the $E_{j}\left(\lambda \right)$ and the α(λ) of each piont are different, and in formula (4), the *L* of each point are different. Therefore, it is impossible to obtain high-precision reconstruction results only by applying traditional multi-spectral photometric stereo algorithms.

### 2.2. Introduction of Photometric Stereo Algorithm Based on Laser Line Correction

**Figure 2:**
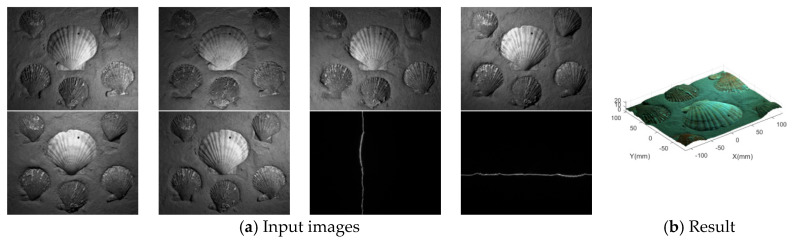
The overview of the Fan’s algorithm. (**a**) are the 8 input images, including 6 gray images which are acquired on 6 different angles, and 2 laser images acquired when the laser source is the only illumination source, respectively, (**b**) is the reconstruction result.

Fan [16] proposed an algorithm, which could obtain the high-precision reconstruction results, through correcting the 3D reconstruction results obtained by the photometric stereo algorithm by laser triangulation. The overview of the Fan’s algorithm is shown in Figure 2.

The reconstruction result of the FAN’s algorithm can achieve high accuracy, but it is required that the camera and the target remain relatively still during the image acquisition process. The motivation of this manuscript is to use deep learning to decompose a multi-spectral image with laser lines into a multi-spectral image without laser lines, and a pure laser line image, and at first calculate the rough 3D shape through the traditional multi-spectral photometric stereo algorithm, then, obtain the height information of the area covered by the laser line through the laser triangulation algorithm, and finally, correct the reconstruction result of the target surface through the algorithm proposed by Fan.

### 2.3. Laser Line Extraction Algorithms

The laser scanning method is one of the earliest proposed and fully studied 3D reconstruction methods, and even commercially available [18], but most of these devices or algorithms can only process the laser line extraction problem when the laser is the only light source or the ambient light is very weak relative to the laser brightness. In terms of algorithmic development in this field, there are many kinds of research studies on laser line thinning. Molder et al. [19] proposed and studied two laser line center position detection methods, which improved the laser line detection results to sub-pixel accuracy. Natalija et al. [20] proposed a new laser line center estimation algorithm. Li et al. [21] proposed the gray gravity method to extract the center of laser stripes. Ta et al. [22] proposed a laser line detection method, using the color space to enhance the laser signal and reduce the discrimination effect of white ambient light, to improve the accuracy of the line detection algorithm. Song et al. [23] proposed a hybrid laser image point extraction algorithm using SVD decomposition in the HSV space of the image. Pavel et al. [24] proposed a laser line extraction algorithm based on color segmentation.

However, the premise of the above algorithm for laser line detection is that the intensity of the background and the laser line is significantly different, such as natural light [22,23], or dark illumination environments [24], which is not applicable to the extraction of laser lines in trichromatic laser images.

### 2.4. Image In-Painting Algorithms Based on Deep Learning

Over the last few years, deep learning techniques have yielded significant improvements in image in-painting. Liu et al. [25] proposed a novel coherent semantic attention (CSA) layer to construct the correlation between the deep features of hole regions, and introduced a consistency loss to guide the CSA layer to learn the VGG features of ground truth, to achieve better in-painting results. Hong et al. [26] designed a learnable fusion block, which predicts an alpha composition map to achieve smooth transition, to implement pixel level fusion in the transition region. Yu et al. [27] proposed a unified feed-forward generative network with a novel contextual attention layer for image in-painting. Then they [28] proposed a generative image in-painting system based on gated convolutions to complete images with free-form mask and guidance. Nazeri et al. [29] proposed a two-stage adversarial model EdgeConnect which comprises of an edge generator and an image completion network. The network can hallucinate edges of the missing region through the edge generator and fill in the missing regions through the image completion network. Zeng et al. [30] proposed an iterative in-painting method using a deep generative model with a feedback mechanism, and a guided up-sampling network to enable generation of high-resolution in-painting results. Huang et al. [31] proposed a method for automatically guiding patch-based image completion using mid-level structural cues. Yang et al. [32] proposed a novel pyramid structure loss is proposed to supervise structure learning and embedding, and an attention mechanism is developed to further exploit the recurrent structures and patterns in the image to refine the generated structures and contents. Liu et al. [33] proposed a mutual encoder-decoder network for image in-painting, and a feature equalization method to make structure and texture features consistent with each other, to remove blur and artifacts caused by inconsistent structure and texture features. Jimmy et al. [34] proposed a novel CNN architecture, which can efficiently equip conventional CNN with the ability to learn translation variant operations for irregularly spaced data, and obtain superior performance on both image in-painting and super-resolution. Li et al. [35] proposed a Recurrent Feature Reasoning (RFR) module, which exploits the correlation between adjacent pixels and strengthens the constraints for estimating deeper pixels, leading to better results with exquisite details.

Although image in-painting algorithms based on deep learning have been greatly developed, these algorithms cannot directly deal with the problem in this article. Cause they are either suitable for blind image in-painting [27,28], or for specific images (such as face images) and images under natural light. There is not an image in-painting algorithm for multispectral images. In addition, most of these existing algorithms need to provide a mask map, or calculate the mask map through interactive operations, but in this article, the laser image (i.e., the mask map) is unknown.

## 3. Method

In this section, the details of the algorithm we proposed will be introduced, which mainly include the architecture of the network, the parameter setting and training information of the Generative Adversarial Network we proposed. We first present the architecture of our network in Section 3.1, and detail the parameter settings, loss function and training process in Section 3.2.

### 3.1. Architecture

We designed an improved Generative Adversarial Network base on the network proposed by Isola [17]. The architecture of the proposed network contains twenty-one layers with different weights, where the generator part is composed of an encoder-decoder network composed of eight encoders and eight decoders, and the discriminator is composed of five convolutional networks. We did not make any changes to the discriminant network, but only improved the generator network.

**Figure 3:**
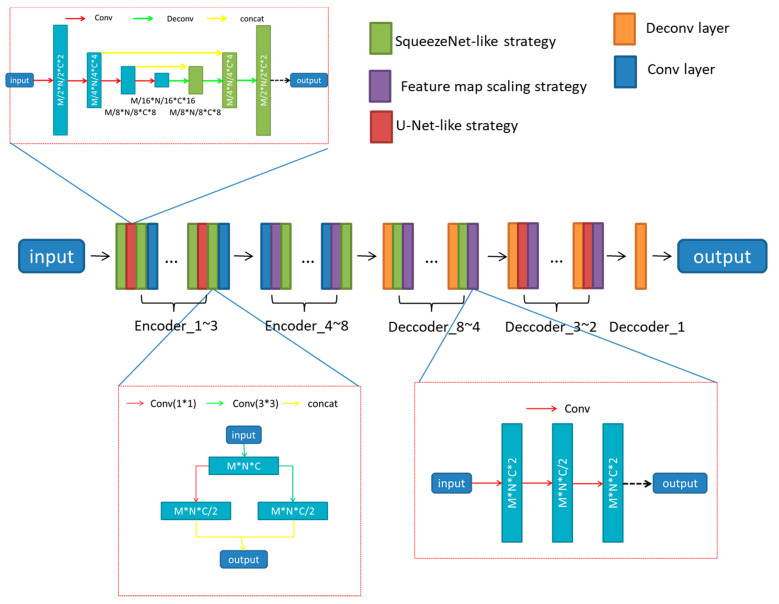
The detailed structure of the generator. M, N, and C are the width, height and channel of the feature map of its input value, respectively.

The architecture of the proposed network is shown in Figure 3, and the details of our generator are expounded in Table 1.

The network proposed by Isola is mainly suitable for tasks such as synthesizing photos from label maps, reconstructing objects from edge maps, and coloring images. That is, the original network will operate on all pixels in the image. In the problem that this manuscript needs to deal with, we hope the network can reduce the operation of pixels in other locations as much as possible while operating the pixels in the area covered by the laser line. Therefore, we have introduced several strategies to realize the attention mechanism so that our proposed network can autonomously determine the location of the area covered by the laser line.

A U-Net-like strategy and a SqueezeNet-like strategy were adopted in the first few layers of the generator to obtain more complete image features, and a scaling strategy of feature map was applied in the other layers of generator to acquire more stable outputs.

U-Net-like strategy The U-Net-like strategy of our network contains four convolutional layers and three de-convolutional layers, with the parameter “striders” set to 2. So the scale of the output of the network is reduced to one-half relative to its input, that is, we use it to replace a convolution strategy with the parameter “strider” is 2, or a simple pooling strategy.

SqueezeNet-like strategy The SqueezeNet-like strategy was used to get more fine-grained features. The strategy contains two convolutional layers, and a concatenation layer, the parameters “kernel_size” of the convolutional layer was set to 1 * 1 and 3 * 3, respectively.

Feature map scaling strategy The Feature map scaling strategy was used to acquire more stable outputs. The strategy contains three convolutional layers.

Novelty As far as we know, no other methods have been found to reduce the scale of feature maps through the U-NET-like strategy, and either the SqueezeNet-like strategy or the strategy which up/down the number of feature map are usually used to classify images. We are the first to use these strategies to solve our problem, and the experiment results prove the effectiveness of our improvement. The verification experiment results are shown in Section 4.3.2.

The improved generator network contains a total of 135 convolution or deconvolution layers. Among them, Encoder 1 to Encoder 3 have 14 layers respectively, including two SqueezeNet-like strategies and one U-NET-like strategy, and a convolutional layer; Encoder 4 to Encoder 8 have 7 layers respectively, Including a SqueezeNet-like strategy, a feature map scaling strategy, and a convolutional layer; decoder 8 to decoder 4, each has 7 layers, including a deconvolution layer, a SqueezeNet-like strategy, and a feature map scaling Strategy, decoder 3 to decoder 2, each has 11 layers, including a deconvolution layer, a U-NET-like strategy, and a feature map scaling strategy. Decoder 1 has only one deconvolution layer.

The input values of all layers should be activated before performing other operations, the activate function for all the generator layers is the Leaky ReLU non-linearity function with the negative slope 0.2, while the activate function of de-convolution layers is ReLU. In decoder_8 to decoder_3, a dropout strategy was adopted with the parameter ‘keep_prob’ set to 0.5. All of these outputs value of the coder-encoder layers in the generator should be batch normalized before output. And in the final layer of decoder, an activation function ‘tanh’ was adopted.

### 3.2. Training

The lack of data makes difficult to train the network with real objects. Inspired by recent advances that use synthetic data for training the deep networks, we also train our network with synthetic images rendered using the ShapeNet dataset. The dataset contains 55 common object categories with about 51,300 unique 3D models. We render the 3D models based on a script on GitHub (https://github.com/panmari/stanford-shapenet-renderer, accessed on 27 November 2017), which can render models to 2D images at different angles with Blender. We have improved the script so that it can generate 2D images at different angles of view for the same target illuminated by the red, green and blue light sources and a laser source.

The loss function was based on conventional l2 residual minimization and that with l1 residual minimization, and their sum was optimized using the Adam Optimizer [36]. We initialize the weights with a zero-mean Gaussian distribution and a standard deviation of 0.02, and the learning rate is set to 0.0005.

## 4. Experiments

In this section, we will evaluate the experimental results of the proposed method, including both rendered and real-world images. All of these results were acquired with a TESLA K40C graphics card.

### 4.1. Dataset

#### 4.1.1. Rendered Image Dataset

We use the Blender Python API to render a total of 13,977 models of five categories in ShapeNet, such as bus, car, airplane, ship and train, into multispectral images with laser lines, multispectral images without laser lines, and laser images.

**Figure 4:**
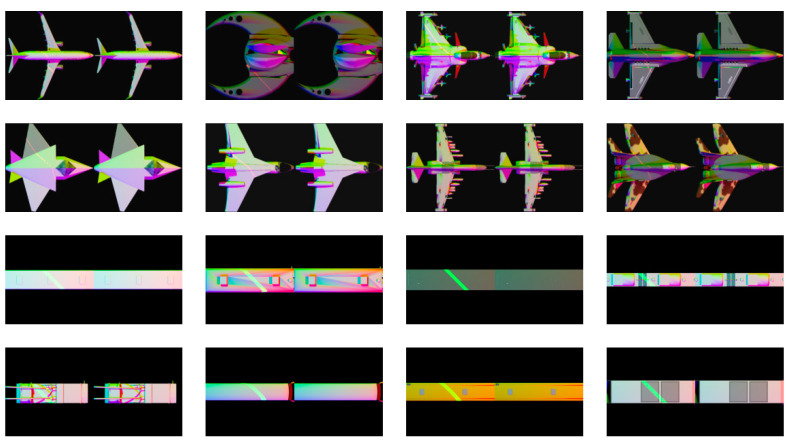
Partly of these rendered images. The left side of each image is a multi-spectral image with laser lines, and the right is a multi-spectral image without laser lines.

The settings were as follows: the resolution of the rendered images was set to 256 * 256; the origin of the coordinate system was set to the center of these models; the lamp type was set to “spotlight”, and their beam were set to 60 degrees, the initial position were set to be at 0 degrees, 120 degrees and 240-degree, the optical axis points to the origin and can move along the ring as a whole at 15 degrees per time; 501 red spotlights were set to simulating the effect of a laser illumination, while their beam was set to 1 degree, the position moved along a straight line, and the color was set to (1,0,0) or (0,1,0), representing red or green laser, respectively. Each model would be rendered to 24 multi-spectral images without laser lines, 24 multi-spectral images with laser lines, and one laser image. Partly of these rendered images are shown in Figure 4.

#### 4.1.2. Real-World Images

We use four objects, including the shell, the ship model, the airplane model and the train model, to create a collection of real-world images at different angles, including multi-spectral images with laser lines, multi-spectral images without laser lines and the pure laser images.

**Figure 5:**
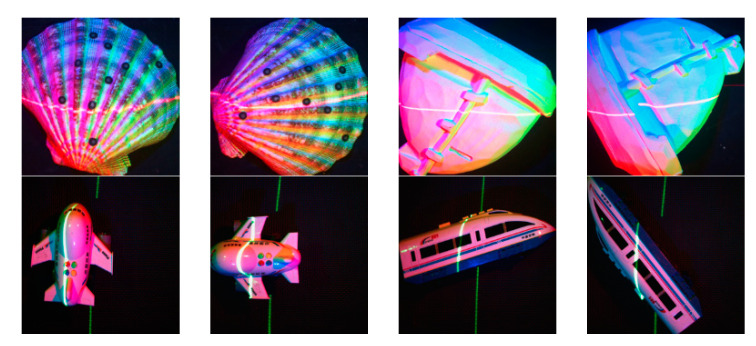
Partly of these real-world images. The images above are multi-spectral images with a red laser line, and the images below are multi-spectral images with a green laser line.

Partly of these real-world images are shown in Figure 5.

### 4.2. Laser Line Extraction Results

We input the multi-spectral image with a laser line into the proposed GAN network to obtain an intermediate result image. After making a difference between it and the input image, we then obtain the desired laser image through threshold segmentation, median filtering, dilation and corrosion. The threshold is set to 0.35 in the experiment.

#### 4.2.1. Extraction Results of Laser Lines in Rendered Images

**Figure 6:**
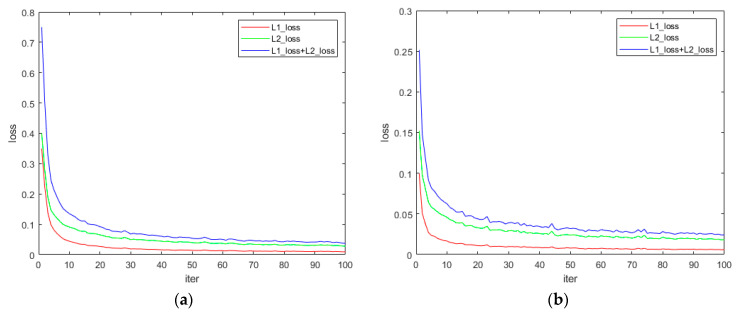
Rendered image training process. (**a**) is the training process of red laser, and (**b**) is the training process of green laser.

First, we input the rendered image into our proposed network for training 100 times, which takes about 5 h with a TESLA K40C graphics card. The training process is shown in Figure 6.

**Figure 7:**
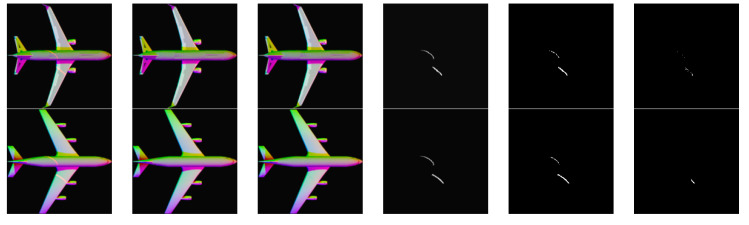

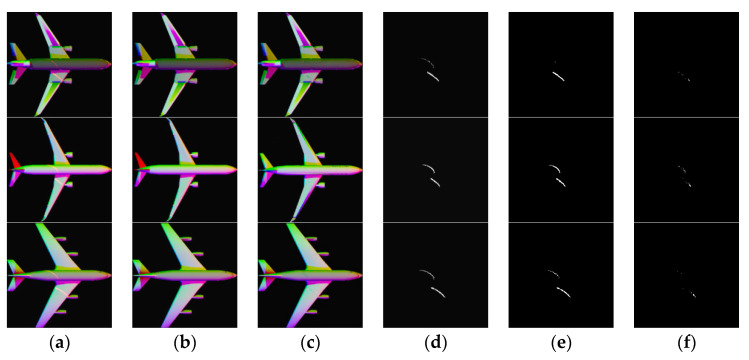
Processing results of rendered images. (**a**) are the input multispectral images with laser lines, (**b**) are the ground truth, (**c**) are the output results of our network, (**d**) are the ground truth of the laser images, (**e**) are the predicted laser images, (**f**) are the laser prediction error images.

The results of laser line extraction of these rendered images are shown in Figure 7.

#### 4.2.2. Extraction Results of Laser Lines in Real-World Images

After training 100 times through the rendered image, three of the real images were input into the network as the training set. After training 500 times, the intermediate result image was obtained. Then the laser image is obtained through thresholding segmentation, median filtering and morphological operations. After experimental debugging, the threshold was set to 0.35 in the experiment.

**Figure 8:**
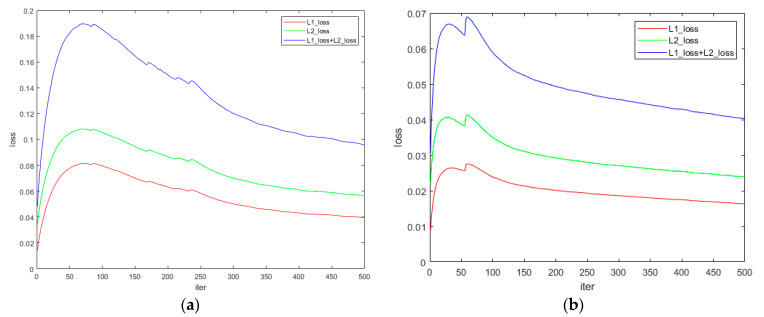
Processing results of real-world images. (**a**) is the training process of red laser, and (**b**) is the training process of green laser.

**Figure 9:**
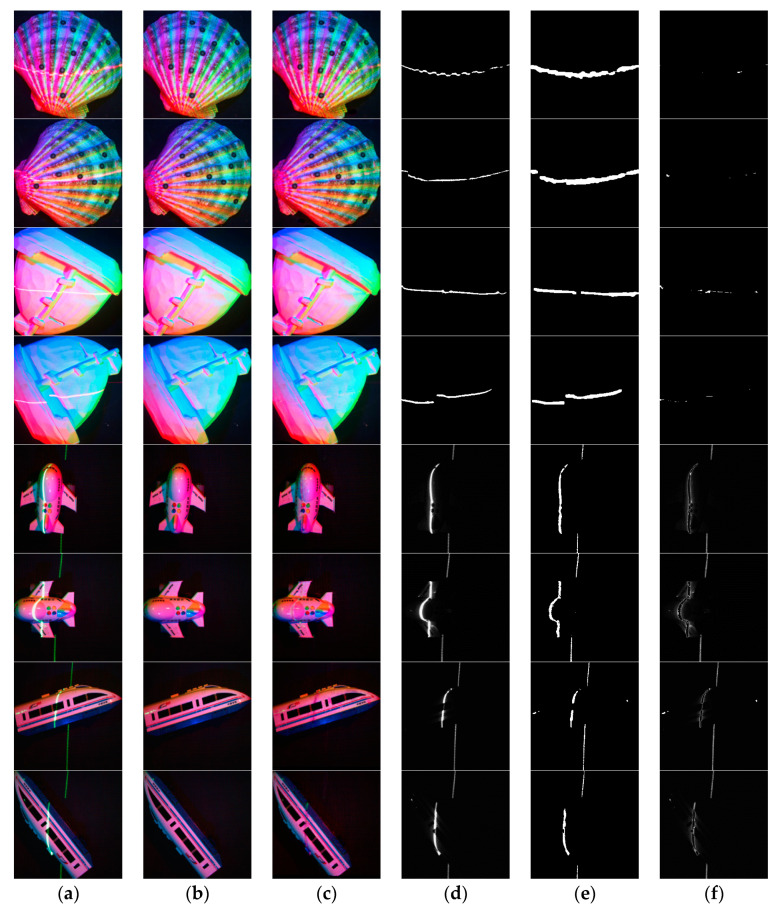
Real-world images processing results. (**a**) are the input multi-spectral images with laser lines, (**b**) are the ground truth, (**c**) are the output results of our network, (**d**) are the ground truth of the laser images, (**e**) are the predicted laser images, (**f**) are the laser prediction error images.

The real image training process is shown in Figure 8, where (a) is the shell training process and (b) is the ship model training process. The laser line extraction results using real images are shown in Figure 9.

In Figure 8, we can see that in the initial stage of training, the loss of the network shows an upward trend, and then slowly declines. This may be because during the training of the rendered images, the network learned to find the position of the laser line autonomously, and estimate the pixel-level RGB values of the pixels covered by the laser line. After the introduction of real images, the network loss increases at the beginning of training because our network uses pixel values as input, and factors such as target’s shape, albedo, and chromaticity will cause changes in pixel values. So, the network needs a small amount of training to interference items such as shape, albedo and chromaticity are eliminated, and regains the capability to find the position of the laser line.

### 4.3. Analysis and Discussion

#### 4.3.1. Comparison with the Results of Isola’s Network

**Figure 10:**
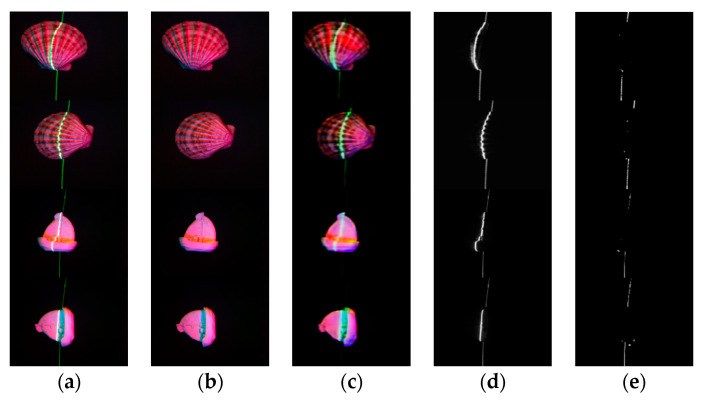
The result of the network proposed by Isola [17]. (**a**) are the input multi-spectral images with laser lines, (**b**) are the ground truth, (**c**) are the output results of the network, (**d**) are the ground truth of the laser images, (**e**) are the predicted laser images of the network.

Comparing the results of our network with the results of the algorithm proposed by Isola [17], the algorithm we proposed can effectively suppress the pixel value in the laser line area, while the network is to suppress all the pixel values in the image, thus the desired result cannot be obtained. These results of the network are shown in Figure 10.

#### 4.3.2. Analysis of the Added Strategy

**Figure 11:**
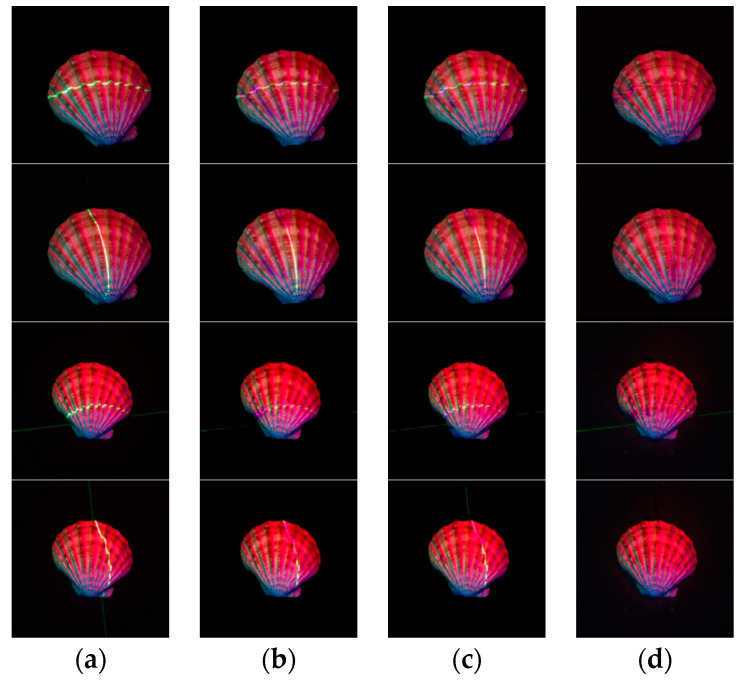
Analysis results of the added strategy. (**a**) are the input multi-spectral images with laser lines, (**b**) are the results when we add the scaling strategy of feature map to the network, (**c**) are the results when we add the scaling strategy of feature map and the SqueezeNet-like strategy to the network, and (**d**) are the results when we add all the three strategies to the network.

Based on the network proposed by Isola [17], we added three strategies, which was a U-Net-like strategy, a SqueezeNet-like strategy, and a scaling strategy of feature map. Figure 11 shows the results when we add one, two and all strategies to the network.

#### 4.3.3. Comparison with Other Image In-Painting Algorithms

**Figure 12:**
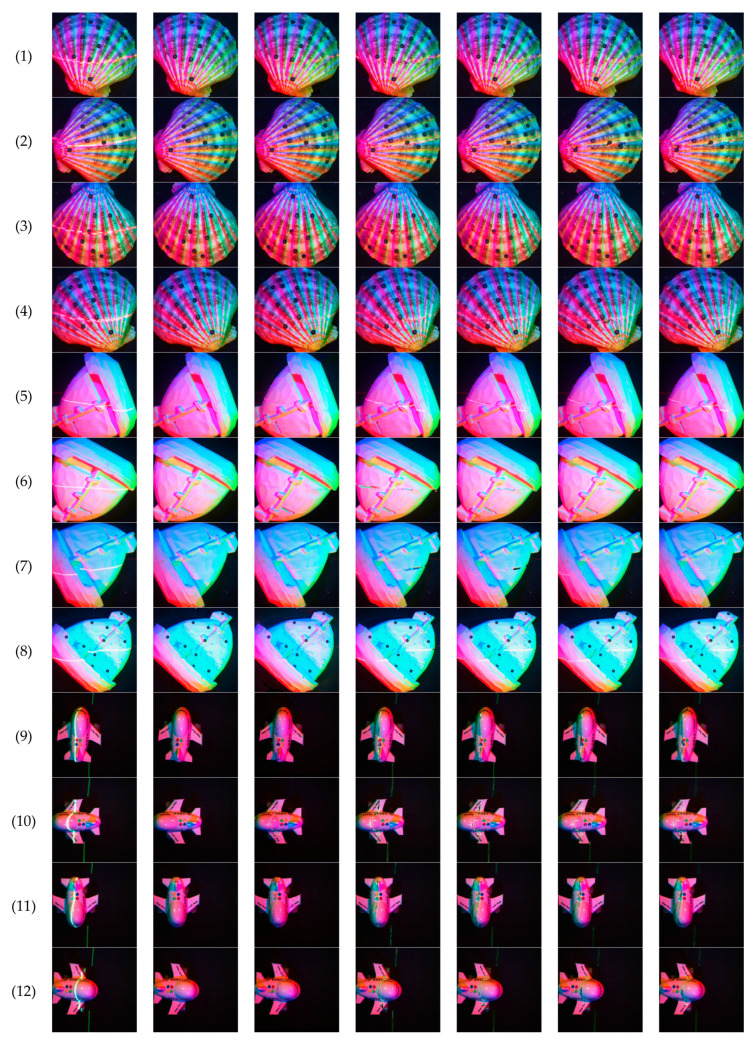

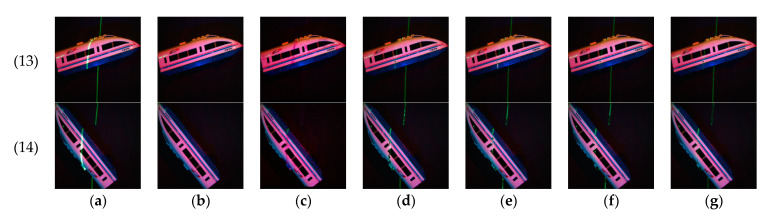
Compares the results of the image completion algorithm proposed by Criminisi (**a**) are the multi-spectral images with laser lines, (**b**) are the ground truth, (**c**) are the output results of our network, (**d**–**g**) are the results of the algorithm proposed in Criminisi’s article, when the parameter “patch_size” is set to 3, 5, 7, and 9.

Comparing the results of our network with the results of the algorithm proposed by Criminisi [37,38], we further verify the advantage of the proposed network. These comparison results are shown in Figure 12 and Table 2, where all the analysis results are compared in the laser line area obtained in the previous step.

Furthermore, we also compared the results of our network with those of Lu’s algorithm [39] and Zeng’s algorithm [30] (http://47.57.135.203:2333/, accessed on 5 January 2021), which are the newest algorithms of image in-painting.

**Figure 13:**
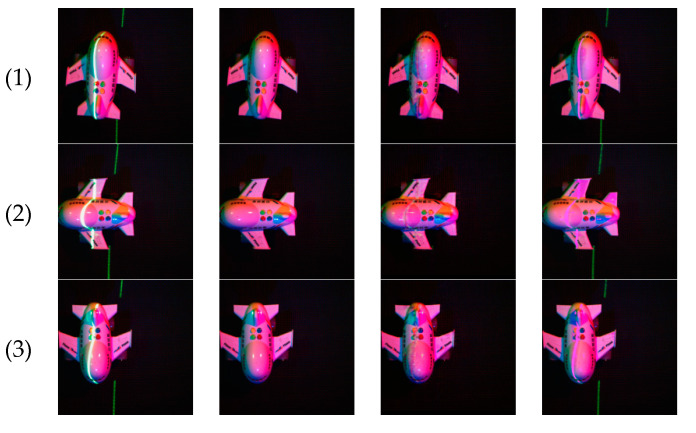

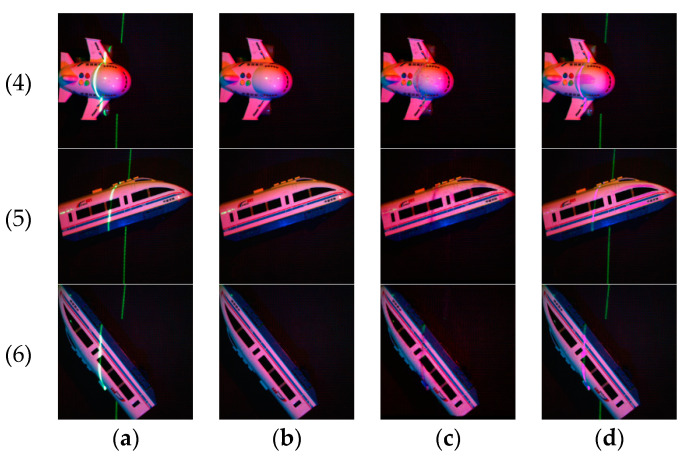
Compares the results of our network with Lu’s algorithm. (**a**) are the multi-spectral images with laser lines, (**b**) are the ground truth, (**c**) are the output results of our network, and (**d**) are the output results of Lu’s algorithm.

The compared the results of our network with those of Lu’s algorithm are shown in Figure 13 and Table 3.

**Figure 14:**
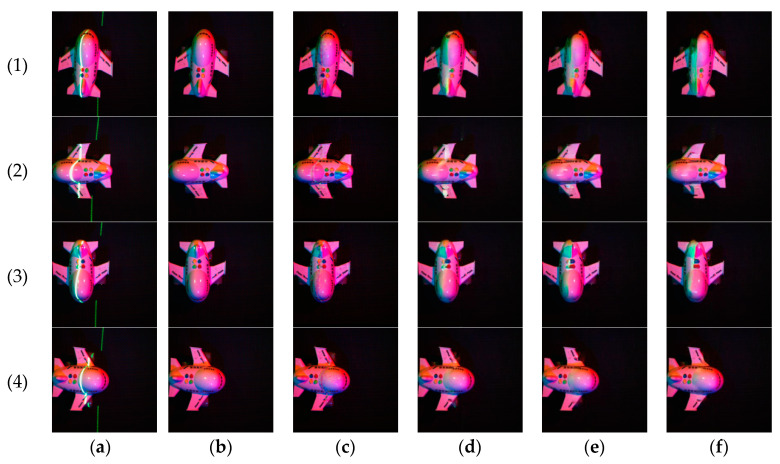
Compares the results of our network with Zeng’s algorithm. (**a**) are the multi-spectral images with laser lines, (**b**) are the ground truth, (**c**) are the output results of our network, and (**d**–**f**) are the output results of Zeng’s algorithm, when the parameter “Line width” is set to 9, 13 and 17.

The compared the results of our network with those of Zeng’s algorithm are shown in Figure 14 and Table 4.

### 4.4. Reconstruction Results Using Multi-Spectral Photometric Stereo

**Figure 15:**
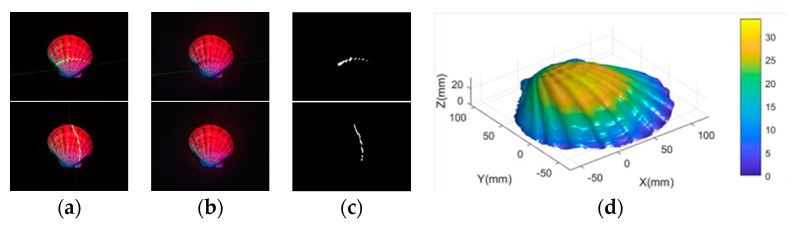
The three-dimensional reconstruction result. (**a**) are the input multi-spectral images with a laser line, (**b**) are the output results of our proposed network, (**c**) are the results of laser line extraction, and (**d**) is the three-dimensional reconstruction result obtained by substituting the above results into Fan’s algorithm.

We input the result of our proposed network into the network of Lu [7] to obtain the predicted depth image and acquire the three-dimensional reconstruction result of the target using the photometric stereo algorithm based on cross laser correction Fan [16]. The result is shown in Figure 15.

## 5. Conclusions

In this work, we have studied the three-dimensional reconstruction algorithm of multi-spectral images with laser lines. The Generative Adversarial Network based on image in-painting has generated laser images and multi-spectral images without laser lines, and realized the three-dimensional reconstruction based on multi-spectral images. A large number of experiments show that the proposed network can effectively extract the laser lines in the multi-spectral images with laser lines, and at the same time, the pixel values of the area covered by the laser lines are also effectively estimated.

However, there is still some room for improvement of the proposed algorithm. For example, in the process of converting the output of the network into a laser image, it is still necessary to set a threshold for morphological processing. In addition, the main application scenario of the algorithm proposed in this article is the three-dimensional reconstruction of underwater images, however, the images which this network can process effectively are acquired in the air, and the result of underwater images in-painting is not ideal. Continue to improve the network to achieve automatic laser line extraction and pixel value estimation for underwater images will be the next goal of our continued research.

## Figures and Tables

**Table 1:** Details of our proposed network.

Name	Input_Channels	Output_Channels
Encoder_1	3	64
Encoder_2	64	128
Encoder_3	128	256
Encoder_4	256	512
Encoder_5~8	512	512
Decoder_8~5	512	512
Decoder_4	512	256
Decoder_3	256	128
Decoder_2	128	64
Decoder_1	64	3

**Table 2:** Compare the results of the image completion algorithm in Criminisi’s article.

Image	MSE of Our Result	MSE of the Result of Criminisi’s Algorithm When the Parameter “patch_size” Is			
		3	5	7	9
(1)	0.0040	0.0073	0.0065	0.0060	0.0057
(2)	0.0037	0.0083	0.0083	0.0094	0.0084
(3)	0.0030	0.0055	0.0050	0.0052	0.0055
(4)	0.0044	0.0068	0.0071	0.0068	0.0060
(5)	0.0035	0.0075	0.0072	0.0066	0.0050
(6)	0.0032	0.0082	0.0057	0.0053	0.0056
(7)	0.0031	0.0079	0.0086	0.0037	0.0025
(8)	0.0059	0.0088	0.0083	0.0078	0.0061
(9)	0.0081	0.0140	0.0123	0.0132	0.0147
(10)	0.0049	0.0125	0.0113	0.0091	0.0104
(11)	0.0066	0.0114	0.0098	0.0096	0.0102
(12)	0.0066	0.0127	0.0102	0.0098	0.0095
(13)	0.0085	0.0092	0.0104	0.0094	0.0090
(14)	0.0076	0.0163	0.0152	0.0131	0.0100

**Table 3:** Compares the results of our network with Lu’s algorithm.

Image	MSE of Our Result	MSE of the Result of Lu’s Algorithm
(1)	0.0081	0.0231
(2)	0.0049	0.0267
(3)	0.0066	0.0259
(4)	0.0066	0.0266
(5)	0.0085	0.0279
(6)	0.0076	0.0307

**Table 4:** Compares the results of our network with Zeng’s algorithm.

Image	MSE of Our Result	MSE of the Result of Zeng’s Algorithm When the Parameter “Line width” Is Set to		
		9	13	17
(1)	0.0081	0.0141	0.0120	0.0131
(2)	0.0049	0.0113	0.0079	0.0082
(3)	0.0066	0.0090	0.0103	0.0113
(4)	0.0066	0.0078	0.0098	0.0093

## Data Availability

Not applicable.
